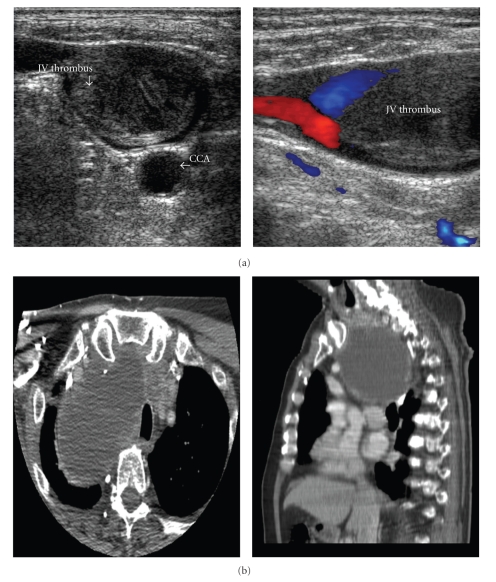# Incidental Detection of Internal Jugular Vein Thrombosis Secondary to Undiagnosed Benign Substernal Goiter

**DOI:** 10.1155/2010/645193

**Published:** 2010-08-11

**Authors:** Mai Tone Lønnebakken, Ole Martin Pedersen, Knut Sverre Andersen, Jan Erik Varhaug

**Affiliations:** ^1^Department of Heart Disease, Haukeland University Hospital, 5021 Bergen, Norway; ^2^Institute of Medicine, University of Bergen, 5020 Bergen, Norway; ^3^Departement of Surgery, Section of Endocrine Surgery, Haukeland University Hospital, 5021 Bergen, Norway

## Abstract

Internal jugular vein thrombosis is a serious event with potentially fatal outcome, where the clinical symptoms may be vague or absent. This paper refers to a rare case where routine carotid Doppler ultrasound prior to coronary artery bypass grafting (CABG) and aortic valve replacement (AVR) in a 76-year-old man, incidentally revealed thrombosis of the right internal jugular vein. Thoracic CT demonstrated an underlying, large, benign substernal multinodular goiter, mainly involving the right lobe, causing compression and displacement of the great vessels. A successful, one-stage operation including ligation of the internal jugular vein to avoid pulmonary embolism and hemithyroidectomy, combined with the scheduled CABG and AVR, was performed. This case illustrates that benign substernal goiter may be associated with asymptomatic internal jugular vein thrombosis. Carotid Doppler ultrasound should involve evaluation of the internal jugular vein concerning thrombosis as its presence may reveal space-occupying lesions in the thorax.

## 1. Introduction

Internal jugular vein thrombosis (IJVT) is a serious event with potentially fatale outcome, including pulmonary embolism as well as intracranial propagation of the thrombus [1]. IJVT is usually caused by damage to the endothelium, alterations of blood flow and hypercoagulopathy [1]. The clinical symptoms may be vague and misleading or absent. However, IJVT may be a part of a superior vena cava (SVC) syndrome, which mostly occurs secondary to malignancy [2, 3]. Compression by a benign, slowly growing, and substernal goiter, leading to stasis and venous thrombosis is rare [4, 5]. However, to the best of our knowledge, this is the first report where routine carotid Doppler ultrasound prior to coronary artery bypass grafting (CABG) and aortic valve replacement (AVR) incidentally detected the combination of IJVT and an underlying huge, asymptomatic, substernal multinodular goiter, and in which all lesions were treated successfully by a one-stage operation. 

## 2. Case Presentation

A 76-year-old man with a medical history of hypertension, lipid disorder, and stable angina presented with sustained ventricular tachycardia and non-ST elevation myocardial infarction. He was without dilatation of the neck veins or any other signs of SVC syndrome. Further examination revealed coronary triple-vessel disease and hemodynamic significant aortic valve stenosis. Routine preoperative carotid Doppler ultrasound was performed. This examination revealed non-compressible intraluminal echoes inside the right internal jugular vein consistent with an intravenous thrombosis, partly adherent to the vessel wall (Figure 1(a)) [6, 7]. Chest X-ray and CT of neck and thorax demonstrated a giant substernal multinodular goiter (8.5 × 11.4 × 9.5 cm) with a dominant right-sided cystic structure, causing compression and displacement of the trachea and the great vessels (Figure 1(b)). Preoperative diagnostic work-up, including laryngoscopy, showed normal thyroid and vocal cord function. Screening for hematologic and oncologic conditions revealed cardiolipin and anti-Beta2-Glycoprotein 1 antibodies in two separate blood tests while lupus anticoagulant was negative.

CABG and biological AVR, combined with right-sided hemithyroidectomy and ligation of the right internal jugular vein were performed in one stage. Surgery started with isolation and ligation of the internal jugular vein in order to prevent dislodgment of thrombotic material from the large vein during and after surgery, which could have caused pulmonary artery embolism. Histopathological examination revealed a benign multinodular goiter with a large, dominantly cystic nodule in the right lobe.

The postoperative course was somewhat prolonged, but without serious complications. He was given acetylsalicylic acid 75 mg once per day and enoksaparin 60 mg once per day as prophylaxis against thrombotic complications. Vocal cord function was normal at laryngoscopy. He was transferred to a local hospital on the 7th postoperative day. On clinical follow-up, two and eight months postoperatively, he was in good health without clinical symptoms or signs of heart- or endocrinological dysfunction. 

Retrospectively, the patient appears to have had an episode of transient swelling of neck and face, and development of superficial varicose veins on the neck some years earlier, which, however, did not lead to any further examination at that time.

## 3. Discussion

The present case shows how routine carotid Doppler ultrasound performed prior to CABG and AVR incidentally detected an IJVT on the right side, leading to CT diagnosis of a huge, undetected, normal functioning, multinodular goiter, mainly involving the right lobe. These lesions were subjected to one-stage surgery, combining CABG, AVR, right-sided hemi-thyroidectomy and ligation of the jugular vein, all performed without serious complications.

In the majority of cases, IJVT is caused by fast-growing infiltrating malignant disease presenting as superior vena cava syndrome due to compression of several mediastinal structures or as a complication to central venous catheterization [2, 3, 8]. Spontaneous IJVT without any predisposing cause is extremely rare, and a predisposing factor is almost always revealed after close examination [1, 9]. Substernal goiter may cause SVC syndrome [10], however, the asymptomatic presentation of a large, benign, substernal goiter detected due to an incidental finding of an IJVT on routine carotid Doppler ultrasound has, to our knowledge, not been described previously.

The cause of the IJVT in this case, substernal goiter mainly involving the right lobe containing a large cyst compressing the great vessels, however, may not be as straightforward as it seems due to (1) prior episode of transient swelling of the neck and face possibly caused by jugular vein thrombosis and (2) current presence of antiphospholipid antibodies, which thus may be part of an antiphospholipid syndrome. Lack of prior episodes of venous thrombosis in other locations than the neck and the rather low predictive value of antiphospholipid antibodies as a single finding, point towards the giant intrathoracic goiter as the most important cause of IJVT in this case [11]. However, the presence of antiphospholipid antibodies may have promoted thrombus formation. The goiter, possibly, has remained undetected for years and thus could have been the cause of the prior episode of symptoms and signs retrospectively connected to IJVT. The slowly growing benign goiter allowing venous collateral formation, in addition to, the dominant cystic nature of the goiter may have caused fluctuations in goiter volume and thus the degree of venous compression from time to time.

Because of the simultaneous presentation of serious coronary artery disease with non-ST elevation myocardial infarction and aortic valve stenosis combined with a giant intrathoracic goiter and IJVT, surgery was performed in one stage, starting with ligation of the thrombosed internal jugular vein in order to prevent per- and postoperative lung embolism [12]. Based upon the uneventful postoperative clinical course, the chosen surgical strategy seems to have been beneficial for the patient. This is also supported by previous publications demonstrating that the risk of pulmonary embolization is low in IJVT [13, 14]. In addition, anticoagulation has not been shown to improve survival in patients with IJVT [14, 15].

## 4. Conclusion

This case illustrates that benign substernal goiter may be associated with IJVT. Although the combination is extremely rare, the silent presentation suggests routine evaluation of the internal jugular vein by ultrasound prior to thyroidectomy in patients with large substernal goiter to prevent per-and postoperative lung embolism. In addition, detection of an IJVT during carotid Doppler ultrasound may be the first sign of a space-occupying lesion in the thorax. If an IJVT is detected by ultrasound, ligation of the thrombosed internal jugular vein should be considered.

##  Disclosure 

No competing interests exist.

##  Consent

Written informed consent was obtained from the patient for publication of this paper and accompanying images.

## Figures and Tables

**Figure 1:**